# Prevalence and molecular detection of *Babesia microti* in rodents in Southeastern Shanxi, China

**DOI:** 10.1371/journal.pone.0306181

**Published:** 2024-07-03

**Authors:** Yiping Liu, Jingrong Niu, Jia Cui, Huaxiang Rao, Juan Yu

**Affiliations:** 1 Department of Basic Medical Sciences, Changzhi Medical College, Changzhi, China; 2 Department of Public Health and Preventive Medicine, Changzhi Medical College, Changzhi, China; University of Bari, ITALY

## Abstract

*Babesia* is a tick-transmitted parasite that infects wild and domestic animals, causes babesiosis in humans, and is an increasing public health concern. Here, we investigated the prevalence and molecular characteristics of *Babesia* infections in the rodents in Southeastern Shanxi, China. Small rodents were captured, and the liver and spleen tissues were used for *Babesia* detection using traditional PCR and sequencing of the partial 18S rRNA gene. The analysis revealed that 27 of 252 small rodents were positive for *Babesia*, with an infection rate of 10.71%. The infection rates in different sexes and rodent tissues were not statistically different, but those in different rodent species, habitats, and sampling sites were statistically different. The highest risk of *Babesia* infection was observed in *Niviventer confucianus* captured from the forests in Huguan County. Forty-three sequences from 27 small rodents positive for *Babesia* infection were identified as *Babesia microti*, including 42 sequences from 26 *N*. *confucianus*, and one sequence from *Apodemus agrarius*. Phylogenetic analysis showed that all sequences were clustered together and had the closest genetic relationship with *Babesia microti* strains isolated from *Rattus losea* and *N*. *confucianus* in China, and belonged to the Kobe-type, which is pathogenic to humans. Compared to other Kobe-type strains based on the nearly complete 18S rRNA gene, the sequences obtained in this study showed the difference by 1–3 bp. Overall, a high prevalence of *Babesia microti* infection was observed in small rodents in Southeastern Shanxi, China, which could benefit us to take the implementation of relevant prevention and control measures in this area.

## Introduction

Since 1888, when *Babesia bigemina* was first identified in the erythrocytes of infected cattle in Romania, more than 120 species of *Babesia* have been identified [1, 2]. *Babesia* species, which are Apicomplexa protozoa belonging to the suborder Piroplasmidea and the family Babesiidae, infect the erythrocytes of a variety of vertebrate hosts, including mammals and some kinds of birds [3]. Several mammals, including dogs, cats, cattle, sheep, cervids, horses and rodents, can be hosts for *Babesia*, become carriers, and are important sources of *Babesia* infections [4–9].

Several species of *Babesia*, including *Babesia microti*, *Babesia venatorum*, *Babesia duncani*, *Babesia divergens* and *Babesia crassa*-*like*, can cause babesiosis in humans [10]. In healthy people infected with *Babesia*, symptoms are generally asymptomatic or mild; however the morbidity and mortality are high in people with severe infection and low immunity. Patients with splenectomies and HIV are at a high risk of *Babesia* infections. The clinical manifestations are intermittent fever, splenomegaly, jaundice, and hemolysis, similar to those of malaria [1]. In recent years, the number of reported human babesiosis cases has been increasing annually, and human babesiosis is considered a significant threat to public health worldwide [11].

*B*. *microti* is the predominant species because of its wide distribution worldwide and increased risk of human diseases. It is a species complex, encompassing at least five distinct clades [12]. Clade 1, referred to as *B*. *microti sensu stricto* (US-type), is a major cause of human babesiosis worldwide. Since the tick *Ixodes dammini* is the main vector, human cases are common only in the United States of America [13]. Clade 2, referred to as *B*. *vulpes*-type, can infect carnivores worldwide, including raccoons, foxes, and badgers [14, 15]. Clade 3, referred to as the Munich-type, has primarily been detected in voles and is prevalent in Europe and North America. The Munich-type is considered to be nonzoonotic [16]. Clade 4, referred to as the Kobe-type, is mainly detected in diverse rodents in Asia, such as Japan, China, and other parts of southeastern Asia, and can also infect humans [17, 18]. Clade 5 comprised the Hobetsu and Otsu types, which have been found primarily in Japan, and no human cases have been reported [18, 19].

Rodents are important hosts of many pathogens and play important roles in the conservation, transmission, and prevalence of diseases [20]. Our previous studies showed that eight species of rodents were found in Southeastern Shanxi, located between the Taihang and Taiyue Mountains, at an average elevation of 900–1000 m. And *Bartonella*, *Borrelia burgdorferi*, *Leptospira interrogans*, *Anaplasma phagocytophilum* and *Orientia tsutsugamushi* were detected in rodents in this area [21–23]. However, investigations of *Babesia* species in the rodents in this area have not yet been reported. In this study, we aimed to investigate the presence of *Babesia* species in the rodents, and explore the molecular characteristics of *Babesia* species in the rodents in Southeastern Shanxi.

## Materials and methods

### Ethical statement

This study was approved by the Ethics Committee of Changzhi Medical College (No: DW2021052). All animals were treated according to the ARRIVE guidelines [24], the Guidelines of Regulations for the Administration of Laboratory Animals (Decree No. 2 of the State Science and Technology Commission of the People’s Republic of China, 1988) and the Guidelines for Treating Animals Kindly from Ministry of Science and Technology of the People’s Republic of China. All efforts were made to minimize discomfort to the animals.

**Fig 1:**
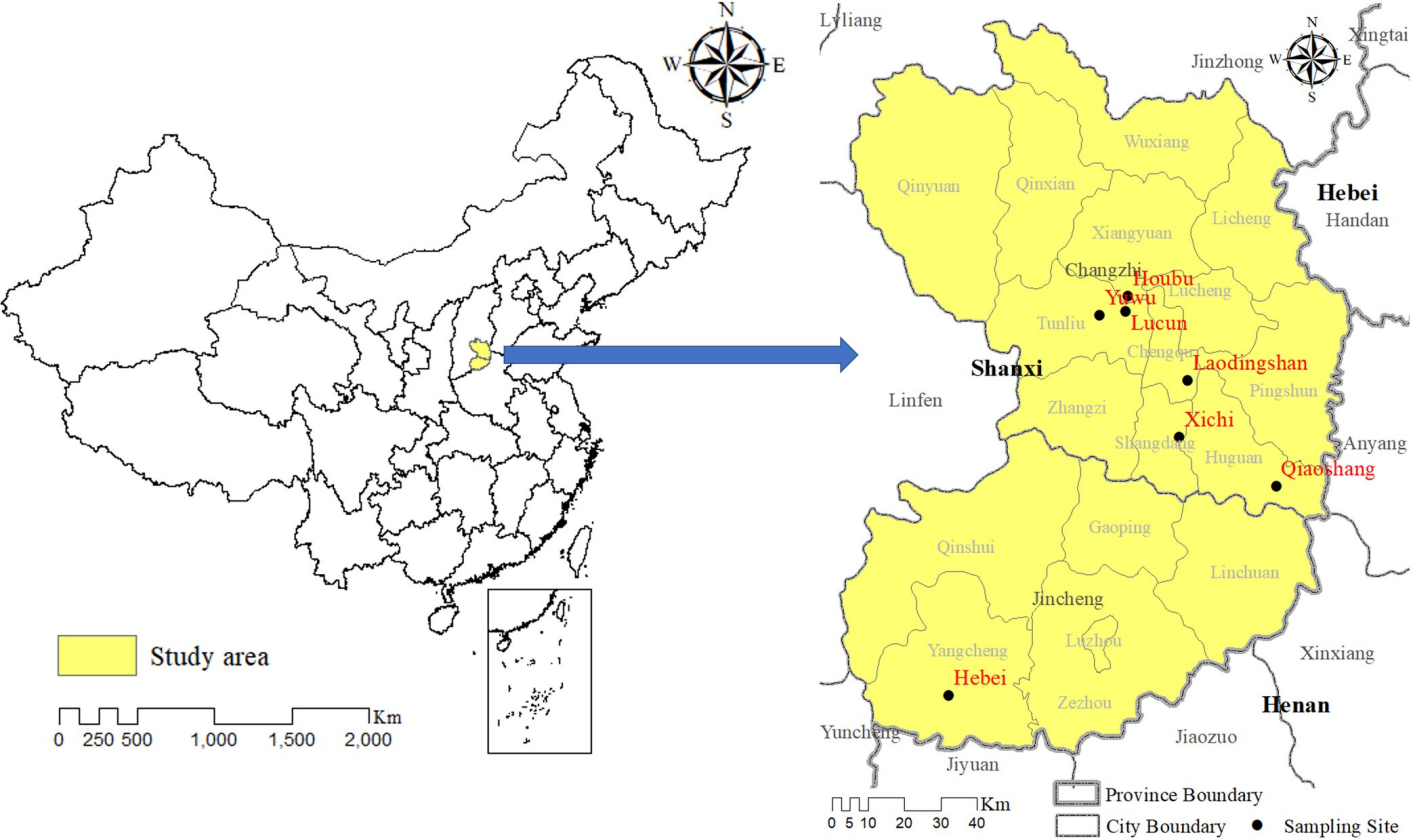
Geographical distribution of the trapped small rodents in the Southeastern Shanxi, China. The map was prepared in ArcGIS 10.2.2 using political boundaries from the National Geomatics Center of China (http://www.ngcc.cn/ngcc) for illustrative purposes only, these data are available free of charge.

**Rodents collection.** In July 2020 and May 2021, small rodents were captured using the night-trapping method [25] in different habitats (villages, farmland, forests and farmland returned to the forest (FRF)). Seven rodents sampling sites were selected randomly, which the distribution was shown in Fig 1. Captured rodents were placed in the transparent plastic box together with degreasing cotton soaked in isoflurane for anesthesia. Euthanasia was performed by cervical dislocation under deep anesthesia, which provide an efficient and quick death that minimizes pain. After euthanasia, the liver and spleen tissues of rodents were then collected and stored at -80°C for later use. The rodents were identified based on their morphology and mitochondrial cytochrome C oxidase subunit I (CO I) gene [21, 22].

#### *Babesia* detection and sequencing

DNA was extracted from approximately 10 mg of liver and spleen tissue according to the manufacturer’s protocols using a TIANamp Micro DNA Kit (TIANGEN Biotech (Beijing) Co., Ltd., China). 18S rRNA gene amplification was performed in 20 μL mixtures containing 10 μL 2×EasyTaq^®^ PCR SuperMix (TransGen Biotech, China), 6.4 μL double-distilled H_2_O, 0.8 μL (10 μmol/L) of each primer (Forward: 5ʹ-GTCTTGTAATTGGAATGATGG-3ʹ, Reverse: 5ʹ-TAGTTTATGGTTAGGACTACG-3ʹ) [26], and 2 μL of DNA template. The amplification was performed under the following conditions: one cycle for 10 min at 94°C; 35 cycles for 30 s at 94°C, 30 s at 55°C, and 45 s at 72°C; and a final extension for 10 min at 72°C. For further confirmation, a nearly full-length 18S rRNA sequences (1198bp) of *B*. *microti* was amplified using nested PCR [27], outer primer 155F 5′- CTAGGGCTAATACATGCTCG-3′ and 1606R 5′- ACTAGGCATTC CTCGTTC-3′ for the first-round PCR and the inner primer 255F 5′-AAATTA GCGAATCGCATGG-3′ and 1453R 5′- ACAGACCTGTTATTGCCTTAC-3′ for the second amplification. Both primary and nested PCR amplifications were performed in a 20 μL mixtures as described above, and 1 μL of the first-round PCR product was used for the second amplification. The conditions for both rounds of amplification were 94°C for 5 min; 35 cycles of 94°C for 30 s, 53°C for 40 s and 72°C for 90 s; followed by a final extension step at 72°C for 7 min. Next, the PCR products were identified by 1.5% agarose gel electrophoresis, and then sent to Sangon Biotech (Shanghai, China) for sequencing.

#### Phylogenetic analysis

The sequences generated in this study were submitted to GenBank (accession numbers: OR492564-OR492590 for partial 18S rRNA, and PP709273 for nearly full-length 18S rRNA). The obtained sequences were blasted against the related *Babesia* species sequences in GenBank using the BLAST program on the National Center for Biotechnology Information Website (http://blast.ncbi.nlm. nih.gov/Blast.cgi). Additionally, 18S rRNA sequences from different *Babesia* species were downloaded from GenBank and used to construct a phylogenetic tree. A neighbor-joining tree was created using the Kimura 2-parameter model in MEGA version 7.0, and bootstrap values were calculated with 1000 replicates [28]. *Plasmodium falciparum* was used as the outgroup.

#### Statistical analysis

The positivity rates of *Babesia* in different sexes and tissues of small rodents were analyzed using the Chi-square test. The positivity rates of *Babesia* in different rodents and rodents captured from different sampling sites and habitats were analyzed using Fisher’s exact test. All data were analyzed using SPSS 22.0 (SPSS, Inc., Chicago, IL, USA). *P* < 0.05 was considered statistically significant.

## Results

### Animal collection

In total, 252 small rodents were captured and classified into eight species, including *Mus musculus* (80), *Apodemus agrarius* (54), *Niviventer confucianus* (48), *Eothenomys inez* (28), *Apodemus draco* (21), *Rattus tanezumi* (17), *Tscherskia triton* (3), and *Apodemus peninsulae* (1).

### Infections of *Babesia* species

*Babesia* was considered positive when at least one tissue sample was amplified using PCR. In total, 27 small rodents were positive for *Babesia* infection, with an infection rate of 10.71% (27/252). *Babesia* was detected in two rodent species (*A*. *agrarius* (1/54) and *N*. *confucianus* (26/48)), but not in the other rodent species, and the difference in the positivity rate among these species was statistically significant (Fisher’s exact test, *P <* 0.001) (Table 1). However, 20 spleen samples were missing during tissue collection, and the positivity rates for the remaining liver and spleen samples were 10.71% (27/252) and 9.48% (22/232) respectively, and there were no significant differences in the positivity rate between these tissues (*χ*^2^ = 0.201, *P* = 0.654) (Table 1). Among the seven sampling sites, *Babesia* infection in rodents in Qiaoshang of Huguan County was higher than that in other areas. The differences in positivity rate among these sampling sites were statistically significant (Fisher’s exact test, *P <* 0.001) (Table 2).

**Table 1:** Positivity rate of *Babesia* infection in different tissues of small rodents.

Host	Liver		Spleen		Total	
	No. detection	No. PCR positive (%)	No. detection	No. PCR positive (%)	No. captured	No. PCR positive (%)
AA	54	1(1.85)	50	0(0.00)	54	1(1.85)
EI	28	0(0.00)	28	0(0.00)	28	0(0.00)
AD	21	0(0.00)	21	0(0.00)	21	0(0.00)
MM	80	0(0.00)	73	0(0.00)	80	0(0.00)
NC	48	26(54.17)	44	22(50.00)	48	26(54.17)
AP	1	0(0.00)	1	0(0.00)	1	0(0.00)
RT	17	0(0.00)	12	0(0.00)	17	0(0.00)
TT	3	0(0.00)	3	0(0.00)	3	0(0.00)
Total	252	27(10.71)	232	22(9.48)	252	27(10.71)

AA: *Apodemus agrarius*, EI: *Eothenomys inez*, AD: *Apodemus draco*, MM: *Mus musculus*, NC: *Niviventer confucianus*, AP: *Apodemus peninsulae*, RT: *Rattus tanezumi*, TT: *Tscherskia triton*.

**Table 2:** Positivity rate of *Babesia* infection of small rodents in different sampling sites.

Sampling sites	Host								No. captured	No. PCR positive	positivity rate (%)
	AA	EI	AD	MM	NC	AP	RT	TT			
Laodingshan	3	0	0	13	0	0	0	0	16	0	0.00
Lucun	6	0	0	8	0	0	2	0	16	0	0.00
Yuwu	0	0	0	34	0	0	3	0	37	0	0.00
Houbu	0	0	0	4	0	0	4	0	8	0	0.00
Xichi	0	0	0	5	0	0	2	0	7	0	0.00
Qiaoshang	13	0	2	2	39	0	5	2	63	25	39.68
Hebei	32	28	19	14	9	1	1	1	105	2	1.90
Total	54	28	21	80	48	1	17	3	252	27	10.71

AA: *Apodemus agrarius*, EI: *Eothenomys inez*, AD: *Apodemus draco*, MM: *Mus musculus*, NC: *Niviventer confucianus*, AP: *Apodemus peninsulae*, RT: *Rattus tanezumi*, TT: *Tscherskia triton*.

Of the 252 small rodents, 106 were males, 146 were females, and the positivity rate was 11.32% (12/106) in males and 10.27% (15/146) in females, which was not statistically significant (*χ*^2^ = 0.070, *P* = 0.791). There were 95 small rodents of four species were captured from the villages, with a *Babesia* infection rate of 1.05% (1/95). Twenty-four small rodents of four species were captured from farmlands, with a positivity rate of 16.67% (4/24). Forty-five small rodents of five species were captured in the forests, with a positivity rate of 44.44% (20/45). Eighty-eight small rodents of seven species were captured in farmland returned to forest (FRF), with an infection rate of 2.27% (2/88). *Babesia* infection rates in rodents from the different habitats were significantly different (Fisher’s exact test, *P <* 0.001) (Table 3).

**Table 3:** Positivity rate of *Babesia* infection of small rodents in different habitats.

Habitats	Host								No. captured	No. PCR positive	positivity rate (%)
	AA	EI	AD	MM	NC	AP	RT	TT			
Village	0	0	1	70	8	0	16	0	95	1	1.05
Farmland	9	0	0	8	6	0	1	0	24	4	16.67
Forest	13	0	1	1	28	0	0	2	45	20	44.44
FRF	32	28	19	1	6	1	0	1	88	2	2.27
Total	54	28	21	80	48	1	17	3	252	27	10.71

AA: *Apodemus agrarius*, EI: *Eothenomys inez*, AD: *Apodemus draco*, MM: *Mus musculus*, NC: *Niviventer confucianus*, AP: *Apodemus peninsulae*, RT: *Rattus tanezumi*, TT: *Tscherskia triton*.

FRF: farmland returned to the forest.

### Identifications of *Babesia* species

**Fig 2:**
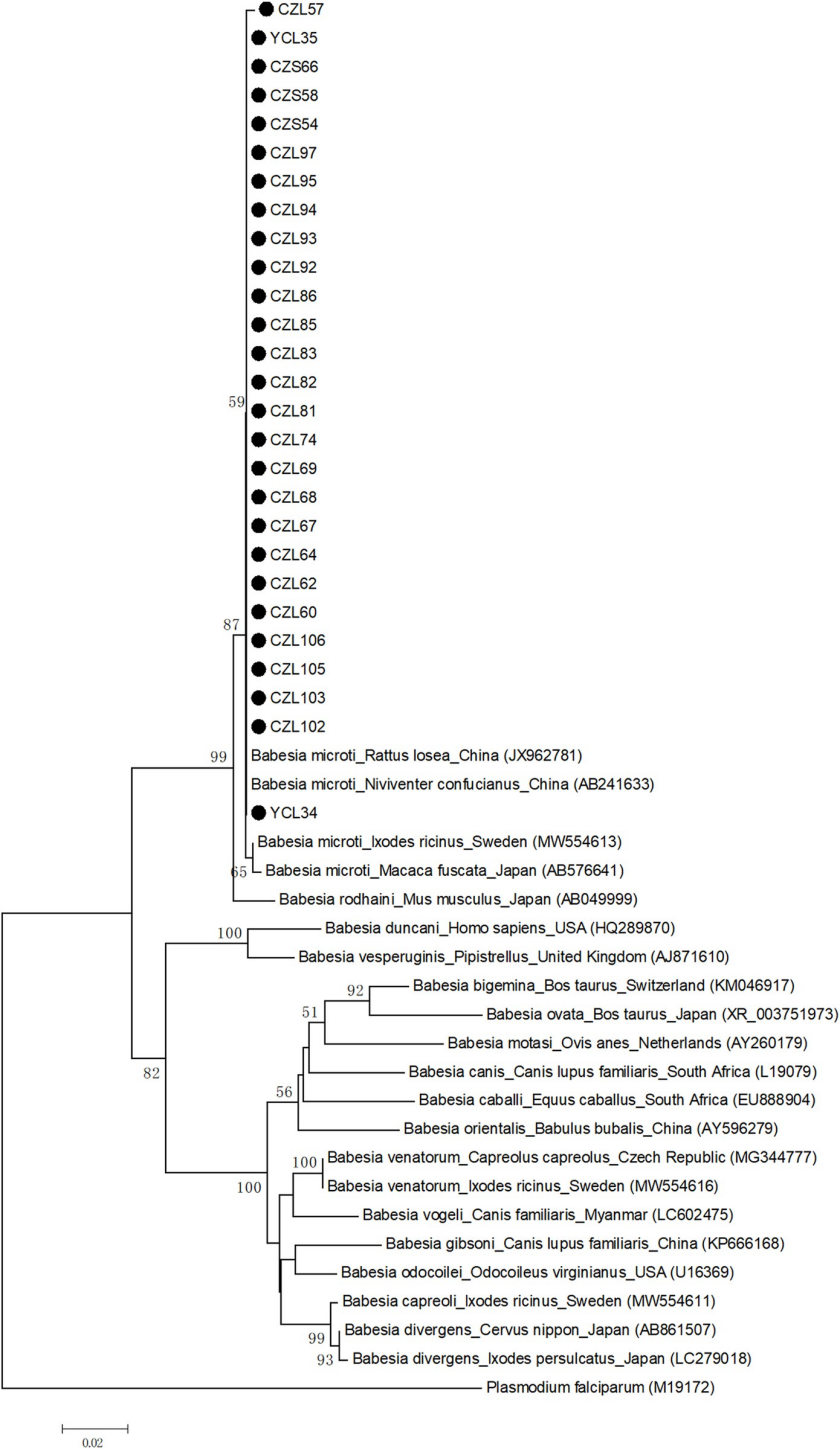
Neighbor joining phylogenetic tree of 18S rRNA gene of *Babesia* species. The tree was constructed by using the neighbour-joining (NJ) method with the Kimura 2-parameter model, bootstrap values calculated with 1000 replicates. Sequences obtained in this study are indicated by black dots. The *Babesia* species, host, region and accession number are given.

**Fig 3:**
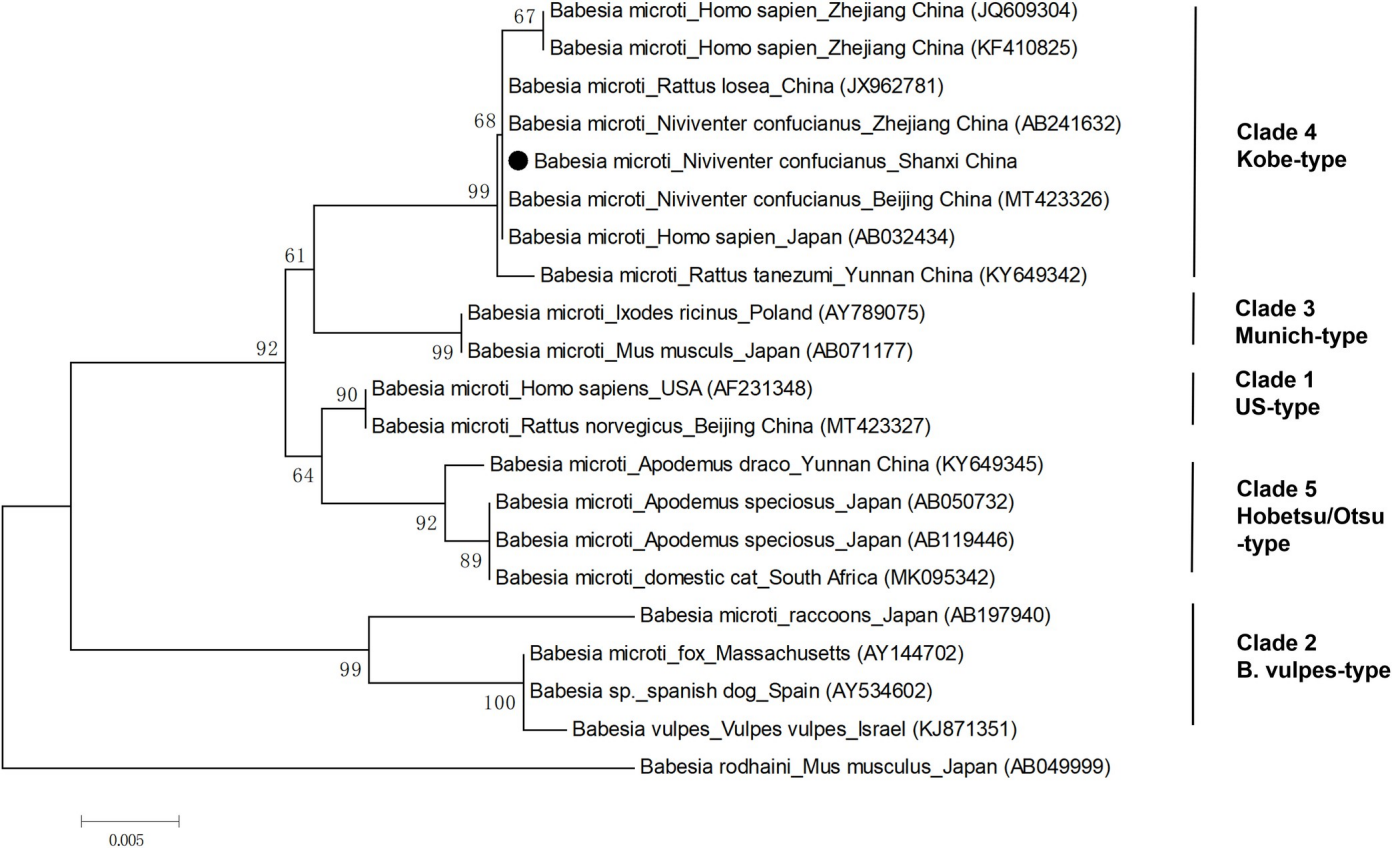
Neighbor joining phylogenetic tree of 18S rRNA gene of *Babesia microti*. The tree was constructed by using the neighbour-joining (NJ) method with the Kimura 2-parameter model, bootstrap values calculated with 1000 replicates. One sequence obtained in this study was selected as the representative, which is indicated by black dots. The *Babesia* species, host, region and accession number are given.

43 sequences from 27 small rodents positive for *Babesia* infection were sequenced the partial 18S rRNA gene successfully. One tissue sample was selected from each rodent for the phylogenetic analysis. The 18S rRNA gene (488 bp) indicated that 27 sequences were *Babesia microti*, including 26 sequences from *N*. *confucianus* and one sequence from *A*. *agrarius*. All the sequences obtained in this study were clustered into a cluster and had the closest genetic relationship with the *B*. *microti* strains isolated from *Rattus losea* and *N*. *confucianus* in China (with 100% identity), which belonged to Kobe-type (Figs 2 and 3). Then, 21 sequences of 18S rRNA gene (1083 bp) were obtained after removing low-quality sequences at both ends, which were identical. We randomly selected one sequence (PP709273) for comparison with other *B*. *microti* strains classified as Kobe-type in Fig 3. The sequence in this study varied by 3 bp from the Kobe-type sequences obtained from a patient in Zhejiang (JQ609304), by 2 bp from a patient in Zhejiang (KF410825) and *Rattus tanezumi* in Yunnan (KT649342), and only 1 bp from other Kobe-type sequences (S1 Fig).

## Discussion

Since the first confirmed case of human babesiosis in Europe in 1957, cases have been reported in Asia, Africa, Australia, Europe, and South America [29]. *B*. *microti* is the main causative agent of human babesiosis in the United States, Canada, and China. However, few cases of *B*. *microti* infection have been reported in Europe [30–32], indicating that there are significant regional differences in *Babesia* infection. Except for *B*. *microti* infection is fatal to immunocompromised individuals, the asymptomatic carriers also pose a threat to recipients when donating blood, an increasing number of transfusion-associated cases of babesiosis are being reported [33]. *B*. *microti* poses an increasing threat to public health worldwide owing to its wide distribution and the increased risk of human infection [34].

Rodents are important hosts of *B*. *microti* and are prone to the transmission of babesiosis owing to their large number, wide distribution, wide range of activities, and many opportunities for contact with humans [35]. *B*. *microti* mainly circulates in a variety of rodent species in many countries, such as those detected in *N*. *confucianus* and *A*. *agrarius* in Southeastern China [17], *A*. *chevrieri* and *N*. *fulvescens* in Northwestern China [27], *A*. *speciosus* and *C*. *rufocanus* in Japan [36], *P*. *leucopus* in the Northeastern United States [36], *M*. *arvalis*, *M*. *agrestis* and *M*. *oeconomus* in Europe [37, 38] and *M*. *agrestis* in Austria [39].

Liver and spleen tissues were used in combination for *Babesia* detection by targeting 18S rRNA gene, which is widely used for *Babesia* identification. The infection rate was not significantly different among the different tissues. However, detection of multiple tissues can improve the positivity rate. The infection rate of *Babesia* species in the rodents was 10.71%, which was similar to that in Henan (9.1% in *R*. *norvegicus*) [40], higher than that in Hangzhou (2.8% in *R*. *tanezumi* and *R*. *norvegicus*) and Xinjiang (1.8% in *C*. *erythrogenys* and *L*. *luteus*) [41], and lower than that in Tiantai Mountain (50% in *N*. *confucianus*) and Fujian (33.3% in *N*. *confucianus*) [17]. *N*. *confucianus* was more prone to harboring the *Babesia* species, which is in accordance with a previous study [17]. This suggests that *N*. *confucianus* is the primary host reservoir in China [42]. The highest risk of *B*. *microti* infection was observed in rodents captured from forests, which may be related to the geographical distribution of *N*. *confucianus* in these natural environments. This indicates that the risk of *Babesia* infection significantly increases when people engage in wildlife activities. In addition, the prevalence of infected rodents in Qiaoshang of Huguan County was high (25/63, 39.68%), which has the national AAAA scenic spot, Taihang Grand Canyon, indicating that local residents and tourists might be at a higher risk for *B*. *microti* infection.

Our study revealed that *B*. *microti* prevalent in this area belongs to the Kobe-type, with the ability to infect humans, which has also been detected in Yunnan, Heilongjiang, Zhejiang, Fujian, and Taiwan[17, 43]. In this study, only one type of *B*. *microti* was observed, which was different from the *B*. *microti* infection in Yunnan, with high genetic diversity [27, 42]. Compared to other Kobe-type strains, the sequences obtained in this study showed a difference of 1–3 bp, suggesting that the 18S rRNA gene of *Babesia* is relatively conserved with little variation.

This study has some limitations in this study: 1) Among the missing spleen specimens, only one corresponding liver specimen was infected with *Babesia*, which would result in a slightly higher infection rate of *Babesia* in the spleen than the actual infection rate. 2) Our results revealed that Huguan County is a high-risk area for *Babesia* infection. We focused only on *Babesia* infection in rodents, the *Babesia* infection in ticks and humans need further investigation.

Our findings provid insights into the prevalence and molecular characteristics of *Babesia* species in small rodents in Southeastern Shanxi, which may aid the prevention and control of babesiosis in this region. It is important to raise awareness of *Babesia* and related tick-borne infectious diseases among people, especially those engaged in forestry and agriculture.

## Conclusion

To the best of our knowledge, this is the first report of *Babesia* infection in rodents in Southeastern Shanxi. Our findings suggest that *B*. *microti* with Kobe-type was detected in two species of rodent, *N*. *confucianus* and *A*. *agrarius*, and *N*. *confucianus* plays an important role in the circulation of *B*. *microti* in this area. Huguan County may be a natural focus of *B*. *microti*, and local residents and tourists should improve their risk awareness and take corresponding preventive measures when engaging in wild activities. Babesiosis, a neglected tick-borne infectious disease, requires further attention.

## Supporting information

S1 Fig Sequence comparision of nearly full-length 18S rRNA gene within Kobe-type *Babesia microti*. (TIF)
