# Supplementary figures and images for: Prevalence and molecular detection of Babesia microti in rodents in Southeastern Shanxi, China
Source: PLoS One. 2024 Jul 3;19(7):e0306181. doi: 10.1371/journal.pone.0306181 (PMC11221649; doi:10.1371/journal.pone.0306181)

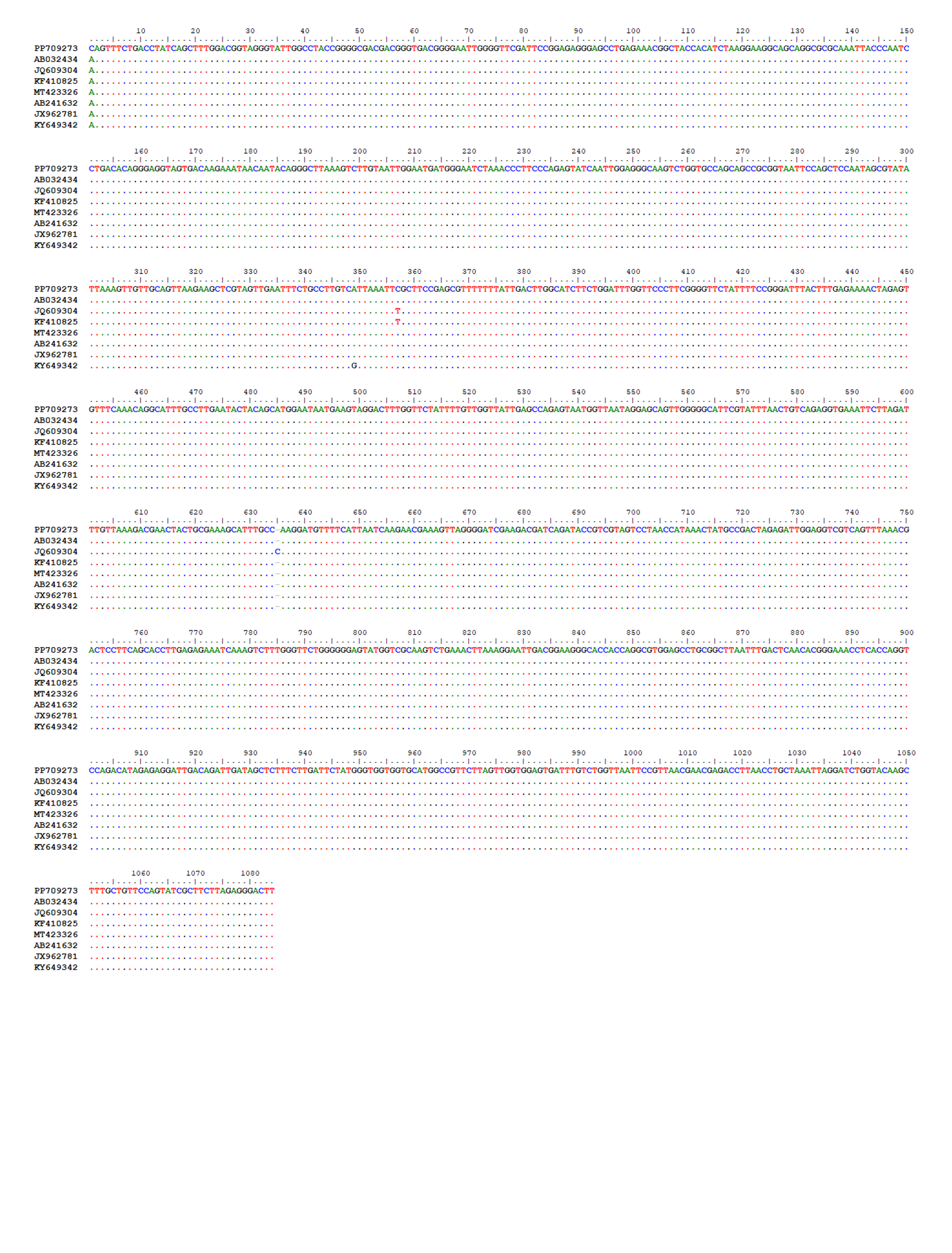

Supplement: S1 Fig — (TIF) [file pone.0306181.s001.tif]
